# Distinct Physiological Roles of Three Phospholipid:Diacylglycerol Acyltransferase Genes in Olive Fruit with Respect to Oil Accumulation and the Response to Abiotic Stress

**DOI:** 10.3389/fpls.2021.751959

**Published:** 2021-11-12

**Authors:** M. Luisa Hernández, Samuele Moretti, M. Dolores Sicardo, Úrsula García, Ana Pérez, Luca Sebastiani, José M. Martínez-Rivas

**Affiliations:** ^1^Department of Biochemistry and Molecular Biology of Plant Products, Instituto de la Grasa (IG-CSIC), Campus Universidad Pablo de Olavide, Seville, Spain; ^2^BioLabs, Institute of Life Sciences, Scuola Superiore Sant’Anna, Pisa, Italy

**Keywords:** *Olea europaea*, olive fruit, oil content, PDAT, gene expression, triacylglycerol synthesis, abiotic stresses

## Abstract

Three different cDNA sequences, designated *OepPDAT1-1*, *OepPDAT1-2*, and *OepPDAT2*, encoding three phospholipid:diacylglycerol acyltransferases (PDAT) have been isolated from olive (*Olea europaea* cv. Picual). Sequence analysis showed the distinctive features typical of the PDAT family and together with phylogenetic analysis indicated that they encode PDAT. Gene expression analysis in different olive tissues showed that transcript levels of these three *PDAT* genes are spatially and temporally regulated and suggested that, in addition to acyl-CoA:diacylglycerol acyltransferase, *OePDAT1-1* may contribute to the biosynthesis of triacylglycerols in the seed, whereas *OePDAT1-2* could be involved in the triacylglycerols content in the mesocarp and, therefore, in the olive oil. The relative contribution of PDAT and acyl-CoA:diacylglycerol acyltransferase enzymes to the triacylglycerols content in olive appears to be tissue-dependent. Furthermore, water regime, temperature, light, and wounding regulate *PDAT* genes at transcriptional level in the olive fruit mesocarp, indicating that PDAT could be involved in the response to abiotic stresses. Altogether, this study represents an advance in our knowledge on the regulation of oil accumulation in oil fruit.

## Introduction

Plant oils are mainly composed of triacylglycerols (TAG), which consist of a glycerol backbone esterified by three fatty acids. TAG constitutes a key storage lipid compound that represents a highly reduced form of carbon to be used as an energy reserve during seed germination and early seedling development (Xu et al., 2018). In addition, TAG are involved in many essential physiological processes such as stress response and pollen germination, since they supply the precursors for lipid signaling and membrane biosynthesis (Yang and Benning, 2018). Plant oils are mainly used for edible applications (food and feed), although there is an increasing interest in their use as renewable raw materials for the production of biofuels, biolubricants, and other bioproducts. For that reason, the global demand for plant oils is rapidly growing (Weselake et al., 2009). To meet this demand, one of the possible strategies is to improve the oil yield in the main oilseed and oil fruit crops (Carlsson et al., 2011). In the oil palm mesocarp, the oil content is about 80% and, therefore, it could be very difficult to achieve an additional increment because a minimum of structural components is necessary to confine this oil within the cell. In seeds, this high oil content could not be reached because they need storage proteins for germination and other cellular components for desiccation. However, in the case of olive mesocarp, which is characterized by an oil content of 40–50%, there is still room for an increase.

Olive is the second most important oil fruit crop cultivated worldwide, with olive oil ranking ninth in the global production of vegetable oils (Rallo et al., 2018). Virgin olive oil is a natural fruit juice increasingly demanded in recent decades due to its exceptional organoleptic properties and potential health benefits (Covas, 2008). Thus, one of the main objectives of the olive breeding programs is the generation of new cultivars, which possess a higher oil content in the olive fruit (Baldoni and Belaj, 2009). However, although new olive cultivars have been generated in the last years, none of them was selected for a higher oil yield despite the first Quantitative Trait Loci (QTLs) controlling oil accumulation in the olive fruit have been reported (Atienza et al., 2014).

In higher plants, TAG are synthesized in the endoplasmic reticulum (ER) by the sequential incorporation of fatty acids in the form of acyl-CoAs into the glycerol backbone (Chapman and Ohlrogge, 2012; Bates, 2016). The final rate-limiting step of this acyl-CoA-dependent pathway, commonly known as the Kennedy pathway, is catalyzed by the acyl-CoA:diacylglycerol acyltransferase (DGAT), which is responsible for the final acylation at the *sn*-3 position of 1,2-diacylglycerol (DAG) to be converted to TAG, using an acyl-CoA as substrate (Lung and Weselake, 2006). DGAT1 enzymes have been mainly related to the accumulation of TAG in oilseeds, whereas DGAT2 shows no sequence homology to DGAT1, and has been involved in the incorporation of unusual fatty acids (Xu et al., 2018).

In the year 2000, a new enzyme called phospholipid:diacylglycerol acyltransferase (PDAT) was discovered, which catalyzes the acyl-CoA-independent synthesis of TAG by transferring an acyl group from the *sn*-2 position of a phospholipid, mainly phosphatidylcholine (PC), to the *sn*-3 position of DAG, yielding TAG and a lysophospholipid (Dahlqvist et al., 2000). The PDAT activity was first characterized in microsomal preparations of the yeast *Saccharomyces cerevisiae* and developing oilseeds (Dahlqvist et al., 2000), although later investigations have confirmed its presence in algae (Yoon et al., 2012; Liu et al., 2016) but not in mammals. In the same study, the first *PDAT* gene was identified from *S. cerevisiae*. Subsequently, two homologs, *AtPDAT1* and *AtPDAT2*, have been identified and characterized from Arabidopsis (Dahlqvist et al., 2000; Ståhl et al., 2004). Later studies indicate that PDAT is encoded by gene families in most plant species (Pan et al., 2015; Falarz et al., 2020).

Concerning its physiological role, PDAT could not be a major determinant of TAG content and composition in developing seeds, since low transcript levels of *AtPDAT1* were observed in Arabidopsis seeds (Ståhl et al., 2004). In addition, there were no significant changes in seed oil content or fatty acid composition in Arabidopsis plants overexpressing *AtPDAT1* (Ståhl et al., 2004) or in an Arabidopsis *pdat1* knockout mutant (Mhaske et al., 2005). However, when *AtPDAT1* was suppressed using RNAi in a *dgat1* mutant background, the seed oil content was reduced by 70–80%, while neither silencing of *AtPDAT2* nor *AtDGAT2* showed a decrease in the oil content compared with the *dgat1* control (Zhang et al., 2009). Accordingly, the expression of *AtPDAT1* is greatly upregulated in seeds of the Arabidopsis *dgat1* mutant, while the *AtPDAT2* and *AtDGAT2* transcript levels were not significantly altered (Xu et al., 2012). Additionally, normal seed and pollen development in the Arabidopsis *pdat1 dgat1* double mutant was disrupted (Zhang et al., 2009). In a more recent study, it has been reported that the MYB96 transcription factor regulates TAG accumulation by activating *DGAT1* and *PDAT1* expression in Arabidopsis seeds (Lee et al., 2018). All these data strongly indicate overlapping roles of PDAT1 and DGAT1 in Arabidopsis seed oil accumulation, which allow PDAT1 to partially compensate for a loss of DGAT1 activity.

In other oilseeds such as *Camelina sativa*, silencing of *PDAT1* using the CRISPR/Cas system (Aznar-Moreno and Durrett, 2017) or microRNA-mediated downregulation (Marmon et al., 2017) brings about a decrease in the linoleic acid content of seed oil, although a reduction in the total oil content was only observed in the first study. These results confirm that DGAT and PDAT enzymes cooperate in the TAG synthesis in oilseeds. Interestingly, the relative *in vitro* activities of both enzymes considerably differed between plant species, and stages of seed development (Banás et al., 2013). Therefore, the relative contribution of PDAT and DGAT enzymes to TAG synthesis is still unclear and needs to be further explored in other oil-bearing and vegetative plant tissues and species (Chapman and Ohlrogge, 2012; Chen et al., 2015).

In some plant species, PDAT exhibits high activity and unique specificity for PC containing unusual fatty acids and channeling them from PC to TAG, to be removed from membrane lipids and sequestered into TAG. For example, *Crepis palaestina* PDAT catalyzes the incorporation of vernoloyl groups into TAG (Dahlqvist et al., 2000), while PDAT1-2 is responsible for the specific transfer of ricinoleoyl groups in castor bean (Kim et al., 2011). In the same way, a specialized PDAT1 has been identified in flax that selectively incorporates α-linolenic acid into TAG (Pan et al., 2013). These PDAT with unique substrate selectivity are mostly expressed in developing seeds and are grouped into a single clade, which is distinct from those of PDAT1 and PDAT2 (Pan et al., 2015).

As opposed to Arabidopsis seeds, overexpression of *AtPDAT1* in Arabidopsis leaves resulted in significant changes in oil content and fatty acid composition, indicating that PDAT1 is a key enzyme for TAG synthesis in this tissue (Fan et al., 2013a). Further studies also revealed a critical physiological role for AtPDAT1-mediated TAG synthesis in the protection against fatty acid-induced cell death in growing tissues (Fan et al., 2013b), and in the process of diverting fatty acids from membrane lipids toward β-oxidation, thereby maintaining membrane lipid homeostasis in Arabidopsis leaves (Fan et al., 2014). In the same tissue, it has also been reported that AtPDAT1 could be involved in stress responses (Fan et al., 2017; Mueller et al., 2017; Demski et al., 2020), although the role of PDAT in plant responses to different stresses remains to be completely elucidated.

Contrary to *AtPDAT1*, *AtPDAT2* seems to be not involved in TAG biosynthesis in Arabidopsis (Zhang et al., 2009), although its transcript level in developing seeds is higher than that of *AtPDAT1* (Ståhl et al., 2004). Similarly, *PDAT2* homologs from castor bean (*RcPDAT2*) and flax (*LuPDAT6*) did not show a substantial role in TAG biosynthesis (Kim et al., 2011; Pan et al., 2013).

Regarding biotechnological applications, castor bean PDAT has been employed to obtain Arabidopsis transgenic plants with increased amounts (up to 25%) of ricinoleic acid in the seed oil by co-expression with castor bean fatty acid hydroxylase (Kim et al., 2011; van Erp et al., 2011, 2015) or lysophosphatidic acid acyltransferase (Lunn et al., 2020). In addition, the α-linolenic acid content of Arabidopsis seed oil was increased up to 10% by seed-specific expression of a flax PDAT (Pan et al., 2013). Furthermore, PDAT overexpression enhanced TAG content of vegetative tissues like in Arabidopsis leaves by up to 7% of the dry weight without affecting membrane lipid composition and plant growth (Fan et al., 2013b, 2019).

Unlike oilseeds, information about the relative contribution of DGAT and PDAT enzymes to TAG biosynthesis in oil fruit is very scarce. From a biotechnological point of view, oil fruit mesocarp possesses the advantage, compared to oilseeds, of altering TAG content and composition without affecting germination rates. In addition, olive fruit constitutes an interesting system to investigate TAG synthesis, because oil accumulation takes place in two distinct regions: the seed, which is enclosed in a woody endocarp, and the mesocarp, with a major impact on the final composition of the olive oil. In the mesocarp, TAG are accumulated to attract animals to aid seed dissemination, while TAG are synthesized in the seed as storage lipids to nourish the embryo during the early steps of germination (Sánchez, 1994). In olive, two *DGAT* genes (*OeDGAT1* and *OeDGAT2*) have been isolated and characterized, which show overlapping but distinct expression patterns during olive mesocarp growth (Giannoulia et al., 2000; Banilas et al., 2011). In contrast, no *PDAT* genes have been characterized in olive to date, although two genes (*OePDAT1-1* and *OePDAT1-2*) have been identified in the olive pollen (Hernández et al., 2020a).

In the present work, the isolation and characterization of three *PDAT* genes in olive are reported. Transcriptional analysis in olive fruit during development and ripening from two distinct olive cultivars was carried out to investigate the physiological role of each *PDAT* gene. In particular, their specific contribution to the oil content in different tissues and their potential involvement in the response to different abiotic stresses in the olive mesocarp were examined.

## Materials and Methods

### Plant Material and Stress Treatments

For tissues and developmental studies, olive (*Olea europaea* L. cv. Picual and Arbequina) trees were grown in the experimental orchard of Instituto de la Grasa, Seville (Spain), with drip irrigation and fertirrigation from the time of full bloom to fruit maturation. Young drupes, developing seeds, and mesocarp tissue were harvested from at least three different olive trees at different weeks after flowering (WAF) corresponding to different developmental stages of the olive fruit: green (9, 12, 16, and 19 WAF); yellowish (23 WAF); turning or veraison (28 and 31 WAF); and mature or fully ripe (35 WAF). Immediately after harvesting, olive tissues were frozen in liquid nitrogen and stored at −80°C. Young leaves were similarly collected.

The study of water deficit was conducted at the Sanabria orchard, a commercial super high-density olive (cv. Arbequina) orchard near Seville (Spain). The full irrigation (FI) and two regulated deficit irrigation (RDI) treatments (60RDI and 30RDI) were applied as defined by Fernández et al. (2013). Olive mesocarp tissue was sampled at different WAF as described by Hernández et al. (2018).

Stress treatments were carried out according to Hernández et al. (2019). Olive branches from Picual and Arbequina cultivars with about 100 olive fruit at turning stage (28 WAF) were collected from different olive trees and incubated in a growth chamber at 25°C with a 12 h light/12 h dark cycle, and a light intensity of 300 μmol m^–2^ s^–1^. These incubation parameters attempted to mimic the physiological conditions of the tree and were considered the standard conditions. For stress treatments, standard conditions were modified depending on the effect studied. For low and high temperature experiments, the branches containing the olive fruit were incubated at 15 or 35°C, respectively, at the standard light intensity. To assess the effect of the darkness, the light was turned off and the standard temperature was maintained. To study the effect of wounding, the whole surface of the olive fruit was mechanically damaged affecting mesocarp tissue, with pressure at zero time using forceps with serrated tips. The zero time of each experiment was selected 2 h after the beginning of the light period to maintain the natural photoperiod day/night of the olive fruit. When indicated, olive mesocarp tissues were sampled, frozen in liquid nitrogen, and stored at −80°C.

### Isolation of Phospholipid:Diacylglycerol Acyltransferase Full-Length cDNA Clones

Candidate olive *PDAT* sequences were found in the olive transcriptome (Muñoz-Mérida et al., 2013) and the olive (var. *sylvestris*) genome (Unver et al., 2017) using the tblastn algorithm together with the amino acid sequences of Arabidopsis *PDAT1* and *PDAT2* genes (Ståhl et al., 2004). Based on these three new sequences, specific pairs of primers for each gene were designed and utilized for PCR amplification with ACCUZYME^TM^ DNA polymerase (Bioline, Spain), which has proofreading activity. An aliquot of an olive Uni-ZAP XR cDNA library constructed with mRNA isolated from 13 WAF olive fruit of cultivar Picual (Haralampidis et al., 1998) was used as DNA template. One fragment with the expected size was generated in each reaction, subcloned into the vector pSpark^®^ I (Canvax, Spain), and sequenced in both directions.

DNA sequencing was performed by GATC (Biotech, Germany). The DNA sequence data were compiled and analyzed with the LASERGENE software package (DNAStar, Madison, WI). The multiple sequence alignments of olive PDAT amino acid sequences were calculated using the ClustalX program and displayed with GeneDoc. Phylogenetic tree analysis was performed using the neighbor-joining method implemented in the Phylip package using Kimura’s correction for multiple substitutions and a 1000 bootstrap data set. TreeView was used to display the tree. The conserved domains in the deduced amino acid sequences were analyzed using the NCBI Conserved Domain Search^1^ and Pfam software.^2^ Prediction on putative N-glycosylation sites was performed using the software NetNGlyc 1.0.^3^ TMHMM analysis was carried out^4^ and subcellular localization was predicted using two different programs: ProtComp 9.0^5^ and TargetP-2.0.^6^

### Total RNA Isolation and cDNA Synthesis

Total RNA isolation was performed as described by Hernández et al. (2005) using 1.5 g of frozen olive tissue. RNA quality verification, removal of contaminating DNA, and cDNA synthesis were carried out according to Hernández et al. (2009).

### Expression Analysis of Phospholipid:Diacylglycerol Acyltransferase Genes

The expression levels of the olive *PDAT* genes were determined by quantitative real-time PCR (qRT-PCR) using a CFX Connect real-time PCR System and iTaq Universal SYBR Green Supermix (BioRad, California, United States) as previously described (Hernández et al., 2019). Primers for gene-specific amplification for *OePDAT1-1, OePDAT1-2*, and *OePDAT2* were designed using the Primer3 program^7^ and the Gene Runner software (Supplementary Table 1). The housekeeping olive ubiquitin2 gene (*OeUBQ2*, AF429430) was used as an endogenous reference to normalize (Hernández et al., 2009). For tissues and developmental studies, the relative expression level of each gene was calculated using the equation 2^–ΔCt^ where ΔCt = (Ct_*GOI*_ − Ct_*UBQ2*_) (Livak and Schmittgen, 2001; Pfaffl, 2004). This method has the advantage of making comparisons at the level of gene expression across developmental stages, cultivars, and genes. Regarding irrigation studies and stress treatments, the qRT-PCR data were calibrated relative to the corresponding gene expression level at 13 WAF from FI treatment and zero time for each stress treatment and cultivar, respectively, as calibrator. In these cases, the 2^–ΔΔCt^ method for relative quantification was followed (Livak and Schmittgen, 2001). The data are presented as means ± SD of three biological replicates, each having two technical replicates per 96 well plate.

### Oil Content Determination

Lipids were extracted as described by Hara and Radin (1978). Fatty acid methyl esters were produced by acid-catalyzed transmethylation (Garcés and Mancha, 1993) and analyzed by gas-liquid chromatography (Román et al., 2015). Heptadecanoic acid was used as an internal standard. Oil content (μg/mg DW) was calculated as the sum of the different fatty acids. Results are presented as means ± SD of three biological replicates, each having three technical replicates.

## Results and Discussion

### cDNA Isolation and Sequence Analysis of Three Olive Phospholipid:Diacylglycerol Acyltransferase Genes

**FIGURE 1:**
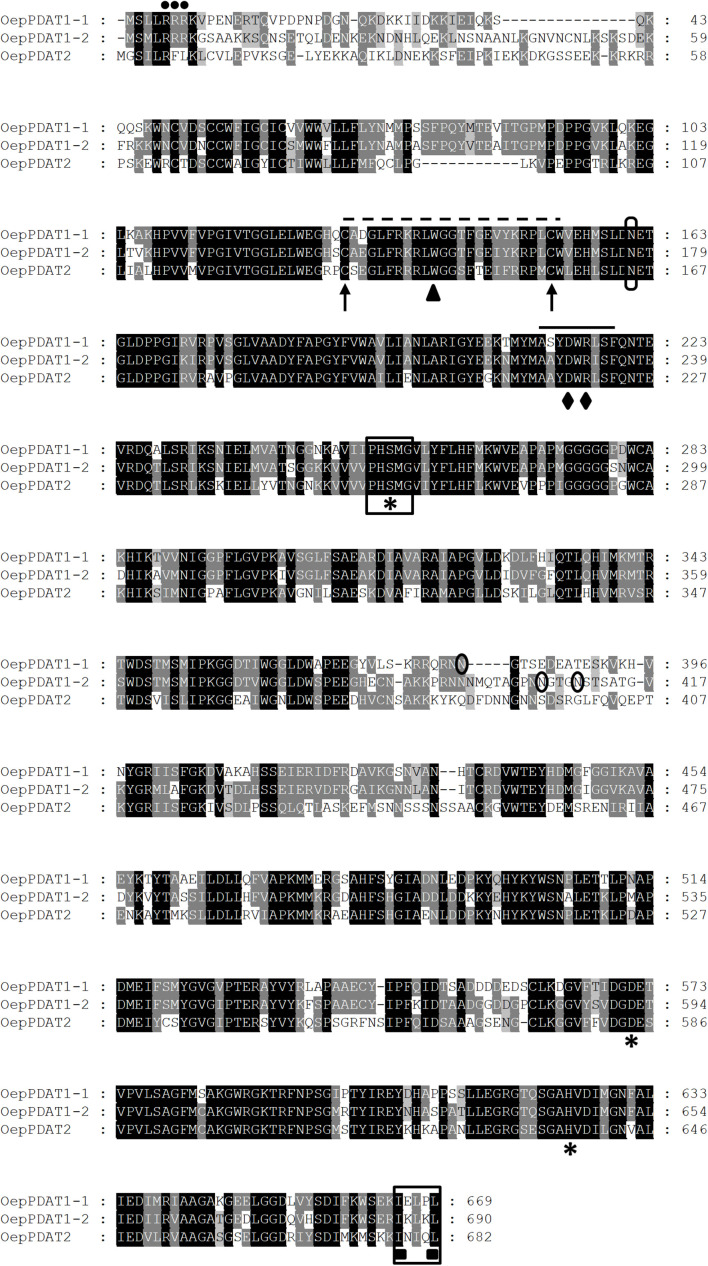
Comparison of the deduced amino acid sequences of olive *PDAT* genes. The sequences were aligned using the ClustalX program and displayed with GeneDoc. Identical and similar residues are shown on a background of black and gray, respectively. The position of the three consecutive Arg residues corresponding to the cluster is marked by dots. The lid domain is identified by a dashed line, flanked by the two conserved Cys residues denoted by arrows and including the conserved Trp residue indicated by a triangle. The conserved domain, which contains a salt bridge between Asp and Arg residues denoted by rhombus, is indicated by a continuous line. The catalytic triad (Ser-Asp-His) is labeled with asterisks and the lipase motif is boxed. The putative N-glycosylation sites are circled. The predicted ER-retrieval motif at the C-terminus of OepPDAT proteins is framed and the two hydrophobic residues are identified with squares. The cDNA sequences corresponding to *OepPDAT1-1, OepPDAT1-2*, and *OepPDAT2* have been deposited in the GenBank/EMBL/DDBJ database with accession numbers MZ614942, MZ614943, and MZ614944, respectively.

Three sequences were selected from the olive transcriptome (Muñoz-Mérida et al., 2013), which exhibited a high degree of similarity to the Arabidopsis *PDAT1* and *PDAT2* genes (Ståhl et al., 2004). These sequences match with the loci LOC111401505, LOC111386227, LOC111374273, and LOC111401783 of the olive (var. *sylvestris*) genome (Unver et al., 2017). Based on these sequences, pairs of specific primers were designed and used for PCR amplification, together with an aliquot of a 13 WAF olive fruit cDNA library (cv. Picual). Three full-length cDNA clones were obtained that were designated as *OepPDAT1-1*, *OepPDAT1-2*, and *OepPDAT2*, with sizes of 2,445, 2,247, and 2,171 bp, respectively. They contained ORFs of 2,007, 2,070, and 2,046 bp, encoding predicted proteins of 669, 690, and 682 amino acid residues, which correspond to calculated molecular masses of 74.7, 76.2, and 76.1 kDa, and p*I* values of 6.1, 7.4, and 8.8, respectively. Alignment of the three olive PDAT deduced amino acid sequences (Figure 1) showed that OepPDAT1-1 displayed 74 and 57% identity with respect to OepPDAT1-2 and OepPDAT2, respectively, while OepPDAT1-2 shared 59% identity with OepPDAT2.

The Conserved Domain search indicated that the three proteins belong to the *PLNO2517* superfamily, with phosphatidylcholine-sterol O-acyltransferase activity. Further characterization of the three olive PDAT using Pfam analysis revealed the presence of a lecithin:cholesterol acyltransferase (LCAT) domain between amino acids 133–630, 149–651, 137–644 for OepPDAT1-1, OepPDAT1-2, and OepPDAT2, respectively, suggesting that they belong to the LCAT superfamily (Pfam:02450).

Several characteristic conserved amino acids and domains were detected in the alignment of the olive PDAT deduced amino acid sequences (Figure 1). A catalytic triad (Ser-Asp-His), which is conserved in all LCAT-like proteins including PDAT (Ståhl et al., 2004). This triad is part of the catalytic domain of LCAT enzymes, in which a fatty acid is transesterified from the *sn*-2 position of phosphatidylcholine to a free hydroxyl group of cholesterol to yield a cholesterol ester (Peelman et al., 1998). Recently, site-directed mutagenesis studies in Arabidopsis PDAT1 have shown that the first residue downstream the Ser of the catalytic triad, a conserved Met, is critical for maintain enzyme activity (Falarz et al., 2020). This Ser residue is also part of a motif analogous to the conserved lipase motif with the consensus sequence Gly-X-Ser-X-Gly (Schrag and Cygler, 1997). In plant PDAT, including the three from olive, the first Gly is replaced with a Pro resulting in the absence of the two β-sheets that may be essential for lipase activity (Yoon et al., 2012). Olive PDAT also contain a so-called lid domain, which is characteristic of lipases and LCAT and is closed by a disulphide bridge. The lid structure occurs between two conserved neighbor Cys residues. This 20–25 (24 for olive PDAT) amino acid long, highly mobile element covers the hydrophobic active site of these enzymes. This lid domain is possibly involved in destabilizing the lipid bilayer, thus facilitating the binding of the hydrophobic substrate and its diffusion into the active site of the enzyme (Peelman et al., 1999; Ståhl et al., 2004). A conserved Trp residue present in this lid domain was predicted to bind the cleaved fatty acid in the active site of these enzymes (Martinelle et al., 1996). In addition, a highly conserved domain that is present in all PDAT sequences contains a salt bridge between Asp and Arg, which may be involved in phospholipid recognition (Yoon et al., 2012), and includes some critical amino acids responsible for its substrate specificity and binding (Peelman et al., 1998). On the other hand, the three olive PDAT deduced amino acid sequences exhibit several potential N-glycosylation sites. Experimental evidence has been recently shown which demonstrates that four Asn residues of the *S. cerevisiae* PDAT protein are glycosylated as a result of a post-translational modification (Feng et al., 2019). However, none of them is conserved in the plant PDAT sequences.

Regarding the membrane topology, transmembrane predictions based on a hidden Markov model (TMHMM) analysis of the olive PDAT proteins were generated (Supplementary Figure 1). Olive PDAT1-1 and PDAT1-2 showed a short hydrophilic N-terminal tail, followed by a putative transmembrane domain (TMD) of 22 amino acids (53–75 and 69–91 for OepPDAT1-1 and OepPDAT1-2, respectively), and the rest of the protein localized in the membrane. This result is in agreement with the topology described for plant PDAT1 proteins (Pan et al., 2015). The hydrophilic N-terminal region preceding the TMD has been reported as the most divergent region, which exhibits a cluster of consecutive Arg residues, as shown in Figure 1 for olive PDAT1-1 and PDAT1-2. The function of these conserved Arg residues remains unknown, although it has been suggested that they are possibly an ER localization signal (Liu et al., 2012). Furthermore, even though the deletion of the TMD together with the N-terminal end of *S. cerevisiae* PDAT does not affect its enzymatic activity and substrate selectivity (Ghosal et al., 2007), the N-terminus of plant PDAT1 could be involved in the sorting of the protein to the ER (Pelham, 2000). Unlike both olive PDAT1 proteins, PDAT2 does not exhibit a TMD (Supplementary Figure 1). Interestingly, except for Arabidopsis PDAT2 (Ståhl et al., 2004), none of the other PDAT2 characterized to date showed a TMD, as it is the case of PDAT2 from *Ricinus communis* (Kim et al., 2011), *Linum usitatissimum* (Pan et al., 2013), *Camelina sativa* (Yuan et al., 2017), and *Sesamum indicum* (Chellamuthu et al., 2019).

Concerning the subcellular localization, the three olive PDAT sequences presented a C-terminal motif (Figure 1), which is both necessary and sufficient for maintaining localization of the enzymes in the ER (McCartney et al., 2004). Similar results have been previously described for Arabidopsis (Ståhl et al., 2004) and *Camelina sativa* (Yuan et al., 2017) PDAT1 and PDAT2. Since the ER is the main site for TAG biosynthesis in plants (Xu et al., 2018), it is assumed that plant PDAT are inserted into the ER and interpret the topology data based on the ER structure. As shown in Supplementary Figure 1, both olive PDAT1 have a short N-terminal end facing the cytosolic side, a single TMD region, and the rest of the protein residing on the lumen side of the ER. This result is consistent with the topology reported for yeast and Arabidopsis PDAT (Ståhl et al., 2004), and with the results reported by Dahlqvist et al. (2000), who detected PDAT activity in yeast and plant microsomal preparations, suggesting a putative localization of the PDAT enzyme in the ER. It is also important to point out that all the conserved motifs and amino acid residues mentioned above are located at the C-terminus of the TMD, indicating that the active and/or binding sites of plant PDAT possibly face the luminal side of the ER (Pan et al., 2015). On the other hand, analysis of the three deduced olive PDAT protein sequences with subcellular localization prediction software such as ProtComp suggests that both olive PDAT1 proteins could be located in the ER, whereas PDAT2 could be localized in the plasma membrane. In line with this observation, *Ricinus communis* PDAT1-1 and PDAT1-2 are located in the ER of epidermal cells of tobacco leaves, while PDAT2 is localized in the plasma membrane (Kim et al., 2011). Moreover, no putative transit peptide was detected in any of the three olive PDAT protein sequences using TargetP software, as previously reported for other plant PDAT (Pan et al., 2015).

**FIGURE 2:**
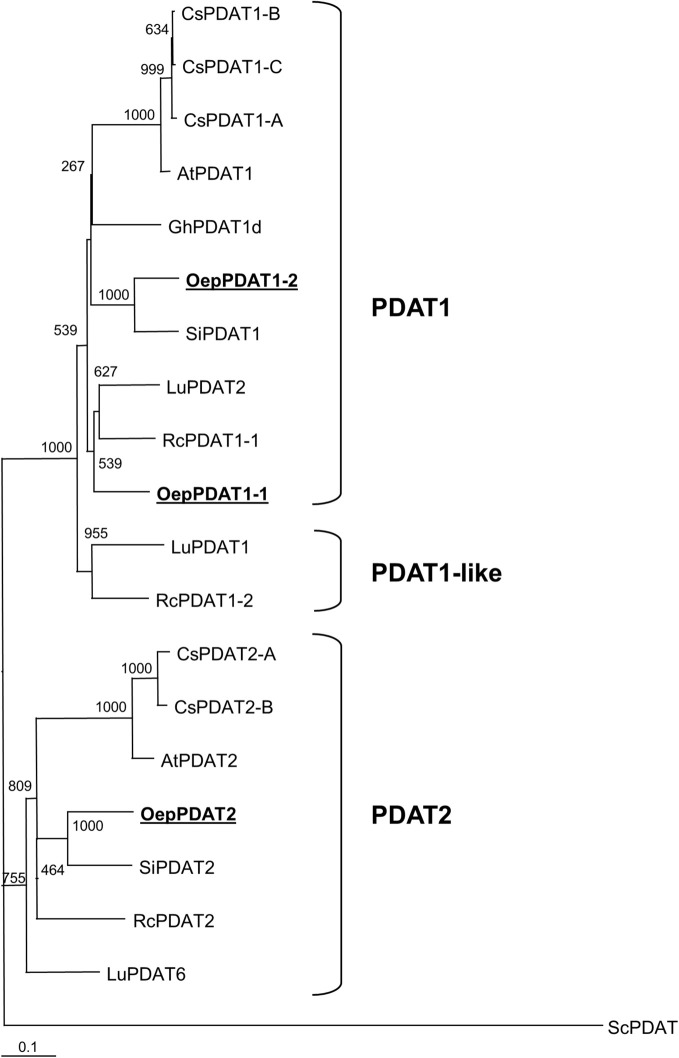
Phylogenetic tree analysis of plant phospholipid:diacylglycerol acyltransferases. Alignments were calculated with ClustalX and the analysis was performed using the neighbor-joining method implemented in the Phylip package using Kimura’s correction for multiple substitutions, and a 1000 bootstrap data set. The *Saccharomyces cerevisiae* PDAT sequence was defined as outgroup. TreeView was used to display the tree. Positions of the three olive PDAT are in bold and underlined. Accession numbers of the different PDAT included in this analysis apart from olive: *Arabidopsis thaliana* (AtPDAT1, AT5G13640; AtPDAT2, AT3G44830); *Camelina sativa* (CsPDAT1-A, XP_010453452; CsPDAT1-B, XP_010419957; CsPDAT1-C, XP_010492131; CsPDAT2-A, XP_010503132; CsPDAT2-B, XP_010514811); *Gossypium hirsutum* (GhPDAT1d, Gh_D09G0766), *Linum usitatissimum* (LuPDAT1, KC437085; LuPDAT2, KC437086; LuPDAT6, KC437087); *Ricinus communis* (RcPDAT1-1, AEJ32005; RcPDAT1-2, AEJ32006; RcPDAT2, AEJ32007); *Sesamum indicum* (SiPDAT1, XP_020553631; SiPDAT2, XP_011088820), and *Saccharomyces cerevisiae* (ScPDAT, NM_001183185).

A phylogenetic tree based on deduced amino acid sequences of all known and characterized algal and plant phospholipid:diacylglycerol acyltransferase was constructed to elucidate the phylogenetic relationship of olive phospholipid:diacylglycerol acyltransferases (Figure 2). In agreement with previous findings, plant PDAT could be classified into three different subfamilies: PDAT1, PDAT1-like, and PDAT2, which correspond to clades VI, V, and VII, respectively, according to Pan et al. (2015). OepPDAT1-1 was positioned close to OepPDAT1-2, in the group of PDAT1 enzymes. On the other hand, OepPDAT2 was situated in the branch together with other plant PDAT2, which show preferential expression in the seed. Interestingly, none of the olive PDAT were placed in clade V, which comprise PDAT enzymes majorly expressed in seeds and with substrate selectivity for unusual or highly unsaturated fatty acids. This is in agreement with the fact that olive oil does not contain unusual fatty acids and exhibits a very low amount of α-linolenic acid.

Collectively, sequence analysis of the three olive PDAT showed the distinctive features typical of the PDAT family and together with phylogenetic analysis indicated that they code for phospholipid:diacylglycerol acyltransferase enzymes.

### Tissue Specificity of Olive Phospholipid:Diacylglycerol Acyltransferase Genes

To investigate the distinct physiological functions of the three olive *PDAT* genes (*OePDAT1-1*, *OePDAT1-2*, and *OePDAT2*), their expression levels were analyzed in olive organs and tissues from the cultivars Picual and Arbequina that exhibit a very active lipid biosynthesis. Specifically, it has been examined young drupes before pit hardening (10–12 WAF), when the seed and mesocarp cells still have not been differentiated and no oil deposition is observed; mesocarp tissue, characterized by the presence of active chloroplasts as well as a strong TAG accumulation; developing seeds, with a high rate of storage lipid biosynthesis; and finally young leaves, where membrane lipid biosynthesis for the photosynthetic machinery is very significant.

**FIGURE 3:**
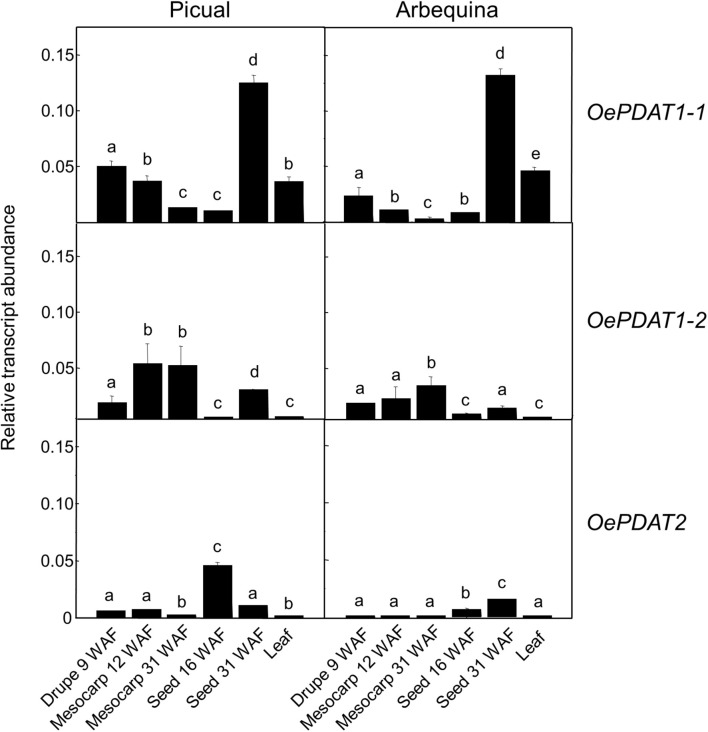
Relative transcript abundance of olive *PDAT* genes in different tissues of Picual and Arbequina cultivars. The relative transcript abundance was determined by qRT-PCR in the indicated tissues as described under section “Materials and Methods.” Data are presented as means ± SD of three biological replicates. Different letters denote significant differences (*P* < 0.05) for each gene and cultivar by one-way ANOVA followed by Tukey’s post-test for multiple comparisons.

As shown in Figure 3, the *OePDAT1-1* gene was highly expressed in young drupes, leaves, and, particularly, in seeds at late stages of development (31 WAF), while *OePDAT1-2* exhibited the highest transcript levels in mesocarp tissue. Regarding *OePDAT2*, expression levels were almost undetectable except in the case of developing seeds, as it has been reported for other plant *PDAT2* genes (Ståhl et al., 2004; Pan et al., 2013). All these data indicate a spatial regulation of *PDAT* genes in olive since they were differentially expressed in all organs and tissues studied. Besides Arabidopsis, the presence of several PDAT isoforms have been described in numerous plant species, each one showing different expression patterns in distinct tissues. Three different *PDAT* genes have been described in castor bean, with *RcPDAT1-1* showing high expression in vegetative tissues, and *RcPDAT1-2* (PDAT1-like) and *RcPDAT2* strongly and predominantly expressed in developing seeds (Kim et al., 2011). Pan et al. (2013) detected six different *PDAT* genes in flax. *LuPDAT2* and *LuPDAT4* (PDAT1 type) were strongly expressed in leaves and other vegetative tissues, whereas *LuPDAT1* and *LuPDAT5* (PDAT1-like), and *LuPDAT3* and *LuPDAT6* (PDAT2 type) were highly and majorly expressed in developing seeds. In *Camelina sativa* five genes have been reported (Yuan et al., 2017). *CsPDAT1-A* and *CsPDAT1-C* exhibited high transcript levels in developing seeds and leaves, respectively, while *CsPDAT2-A* and *CsPDAT2-B* showed low expression in both cases. By contrast, *CsPDAT1-B* was poorly expressed in all organs and tissues studied. In the case of *PDAT1* and *PDAT2* genes from sesame, both displayed high transcript levels in developing seeds and low in leaves and other vegetative organs (Chellamuthu et al., 2019).

In young drupes and leaves, majorly *OePDAT1-1* transcripts were detected. Low levels of expression have been reported for *OeDGAT1* in young drupes, while *OeDGAT2* transcripts were not observed (Banilas et al., 2011). According to our results, *OePDAT1-1* could be also involved together with *OeDGAT1* in the synthesis of the very low amount of TAG present in this organ. In the case of leaves, relatively high transcription levels of both *OeDGAT* genes were observed (Banilas et al., 2011). Therefore, our expression data suggest that *OePDAT1-1* could participate together with *OeDGAT1* and *OeDGAT2* in TAG synthesis in olive leaves. In Arabidopsis leaves, contradictory conclusions have been obtained in different studies regarding the relative contribution of DGAT1 and PDAT1 to TAG synthesis. Comparison of leaf TAG content in the wild type with that of *dgat1* and *pdat1* mutants indicated that in Arabidopsis leaves PDAT1 plays a more essential role than DGAT1 in TAG synthesis (Fan et al., 2013a). On the contrary, *in vivo* labeling experiments showed that [^14^C]12:0 was incorporated into TAG by leaves of the *dgat1* mutant at a much lower rate than that of the *pdat1* mutant, suggesting that DGAT1 is the preponderant enzyme in Arabidopsis leaves responsible for TAG synthesis (Tjellström et al., 2015). Nevertheless, medium chain fatty acids are poorly incorporated into the *sn*-2 position of PC, the acyl donor substrate of PDAT. Thus, it is probable that both DGAT and PDAT are involved in TAG synthesis in leaves, with the relative participation of each enzyme depending on the substrate and conditions of acyl flux (Bates, 2016).

### Developmental Expression of Phospholipid:Diacylglycerol Acyltransferase Genes in the Olive Fruit in Relation to the Oil Content

Next, the transcript levels of olive *PDAT* genes in developing seeds and mesocarp tissue as well as the oil content in the course of fruit development and ripening from the cultivars Picual and Arbequina were examined in more depth, to investigate their specific potential contribution to TAG synthesis in these two oil-accumulating regions from the olive fruit.

**FIGURE 4:**
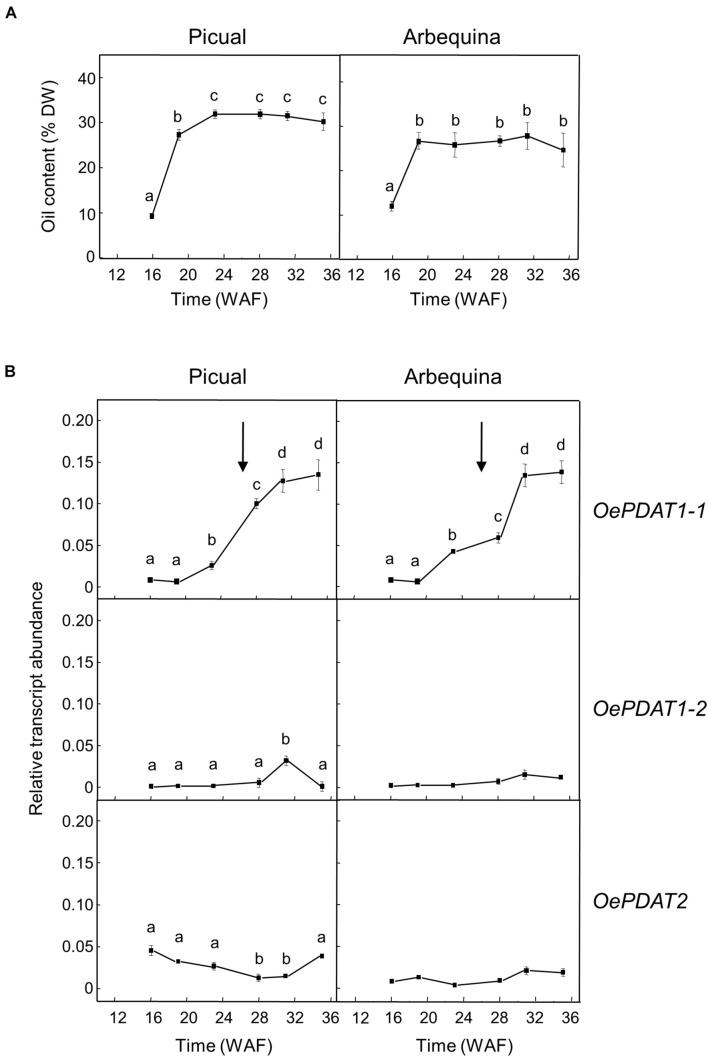
Time-course during olive fruit development and ripening of the oil content **(A)** and the relative transcript abundance of olive *PDAT* genes **(B)** in the seed tissue from Picual and Arbequina cultivars. The beginning of fruit ripening, corresponding to the appearance of pink-purple color, is denoted by an arrow. The oil content and the relative transcript abundance were determined at the indicated stages of fruit development as described under section “Materials and Methods.” Data are presented as means ± SD of three biological replicates. Different letters denote significant differences (*P* < 0.05) for each gene and cultivar by one-way ANOVA followed by Tukey’s post-test for multiple comparisons.

Concerning developing seeds, the oil content rapidly increased from the initial stages, reaching a plateau at around 24 WAF in Picual and Arbequina cultivars (Figure 4A). Gene expression analysis revealed that *OePDAT1-2* and *OePDAT2* exhibited in both cultivars low and constant transcript levels during the whole period of olive fruit development and ripening (Figure 4B). Therefore, none of these two genes seems to be involved in the oil accumulation in olive developing seeds. On the contrary, *OePDAT1-1* showed a significant increase in its expression levels at the onset of ripening of Picual and Arbequina cultivars (Figure 4B), suggesting that this gene could contribute to the oil seed content. However, it is important to point out that the observed increase in *OePDAT1-1* transcript levels occurred when the oil content is constant at later stages of seed development, questioning the possible participation of this *PDAT* gene in TAG synthesis in olive developing seeds.

In oilseeds such as flax, the highest expression of *PDAT* genes in the seed was reported at the early stages of development during the phase of active oil accumulation (Pan et al., 2013). However, the *Camelina sativa CsPDAT1-A* and *CsPDAT1-C* genes augmented their transcript levels in the early and late stages of seed development, respectively, demonstrating that both genes can differently contribute to TAG synthesis (Yuan et al., 2017). In the case of the sesame *PDAT* genes, they showed a continuous increment of expression levels during seed development (Chellamuthu et al., 2019).

In olive developing seeds, the *OeDGAT1* gene has been reported to show a bell-shaped expression pattern, with a peak at 19 WAF and a substantial reduction at 22 WAF, which is similar to that reported for the oleosin gene (Giannoulia et al., 2007). In contrast, *OeDGAT2* expression levels were weak throughout the complete development period (Banilas et al., 2011). Therefore, *OeDGAT1* seems to be responsible for the early and fast oil accumulation observed in olive developing seeds, with a putative contribution of *OePDAT1-1* to TAG synthesis in the final stages of seed development.

**FIGURE 5:**
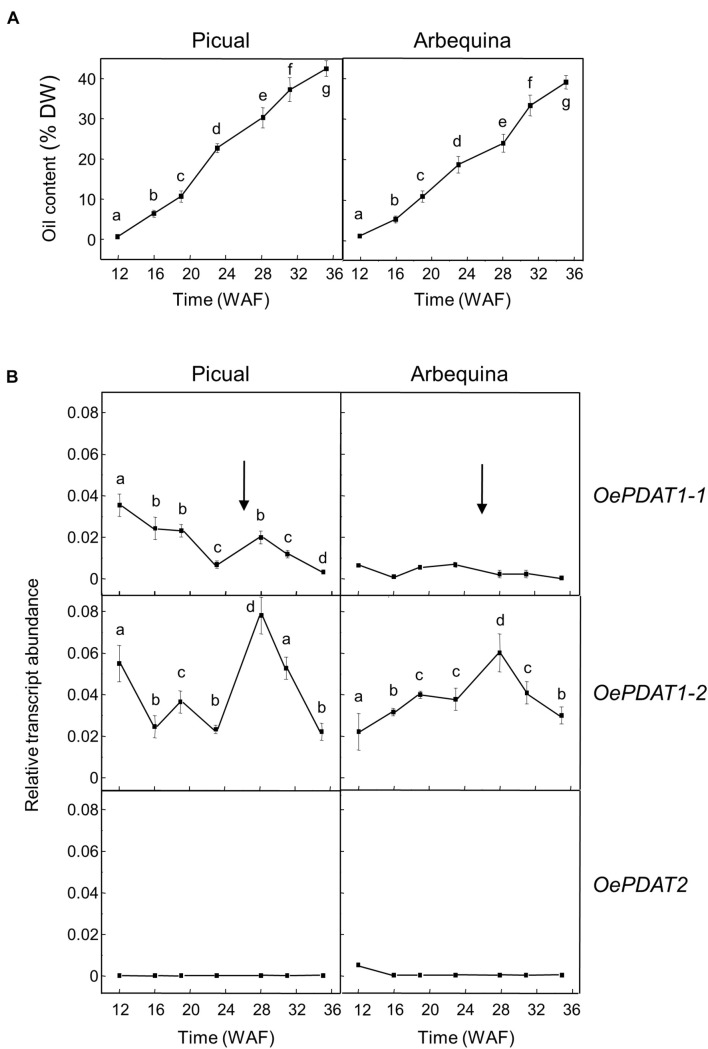
Time-course during olive fruit development and ripening of the oil content **(A)** and the relative transcript abundance of olive *PDAT* genes **(B)** in the mesocarp tissue from Picual and Arbequina cultivars. The beginning of fruit ripening, corresponding to the appearance of pink-purple color, is denoted by an arrow. The oil content and the relative transcript abundance were determined at the indicated stages of fruit development as described under section “Materials and Methods.” Data are presented as means ± SD of three biological replicates. Different letters denote significant differences (*P* < 0.05) for each gene and cultivar by one-way ANOVA followed by Tukey’s post-test for multiple comparisons.

A similar study was conducted in the olive mesocarp (Figure 5). Unlike olive seed, the oil accumulation in the mesocarp of both cultivars gradually increased from the beginning of the fruit development and continued during the ripening period until reach a higher oil content than the seed (Figure 5A). The expression analysis of *PDAT* genes in the mesocarp of Picual and Arbequina cultivars showed low transcript levels and a gradual decrease during the developmental phase for *OePDAT1-1*, while *OePDAT2* expression was almost undetectable (Figure 5B). Conversely, a bell-shaped pattern was detected for *OePDAT1-2* expression levels in both cultivars, showing a maximum at 28 WAF (Figure 5B), which can be associated with the mentioned increment in mesocarp oil content observed during the ripening phase (Figure 5A). These data suggest that the olive *PDAT1-2* gene could contribute to TAG synthesis in the mesocarp when the fruit is in the course of the ripening period. Interestingly, this increase of *OePDAT1-2* transcript levels detected at the onset of ripening is parallel to that reported for the linoleic acid content in the same period for both cultivars (Hernández et al., 2009). These data indicate that the *OePDAT1-2* gene could be involved in the transfer of the linoleic acid synthesized *de novo* in the *sn*-2 position of PC by the microsomal oleate desaturase (FAD2) to the *sn*-3 position of DAG to yield TAG during the ripening period. However, fatty acid analysis of lipid classes during olive mesocarp development and ripening suggested that the incorporation of linoleic acid into TAG might preferentially occur via the Kennedy pathway, with a minor contribution of PDAT activity (Hernández et al., 2020b).

In the olive mesocarp, *OeDGAT1* and *OeDGAT2* genes have been reported to show distinct expression patterns (Banilas et al., 2011). *OeDGAT1* exhibited high transcript levels at the later stages of fruit development (19–25 WAF), with *OeDGAT2* increasing its expression levels at the onset of fruit ripening. Thus, including our results, *OeDGAT1* could be involved in TAG synthesis in the course of the developmental period with a minor contribution of *OePDAT1-1*, whereas *OeDGAT2* and *OePDAT1-2* may have overlapping roles for oil accumulation during the ripening phase.

All of these data also indicate a temporal regulation of the expression of *PDAT* genes in the olive fruit. Moreover, the fact that both, seed and mesocarp, have at least two genes involved in TAG synthesis could be to guarantee oil deposition throughout the long period of development and ripening of the olive fruit, which takes about 35–40 weeks (Sánchez, 1994). Furthermore, since the TAG content of the mesocarp, with a minor contribution of the seed, determines the total TAG content of the olive fruit, *OeDGAT1, OeDGAT2*, and *OePDAT1-2* seem to be the genes mainly responsible for the olive oil content of the fruit.

In summary, all these results suggest that the relative contribution of PDAT and DGAT enzymes to TAG synthesis in olive seems to be tissue-dependent. Supporting this hypothesis, previous studies from our group indicated that PDAT activity may also participate in the TAG biosynthesis in olive callus culture (Hernández et al., 2008), while during pollen germination and pollen tube growth the contribution of DGAT1 but not DGAT2, PDAT1-1 and PDAT1-2 to *de novo* TAG synthesis has been reported (Hernández et al., 2020a).

### Effect of Regulated Deficit Irrigation on Phospholipid:Diacylglycerol Acyltransferase Gene Expression in the Olive Fruit Mesocarp

**FIGURE 6:**
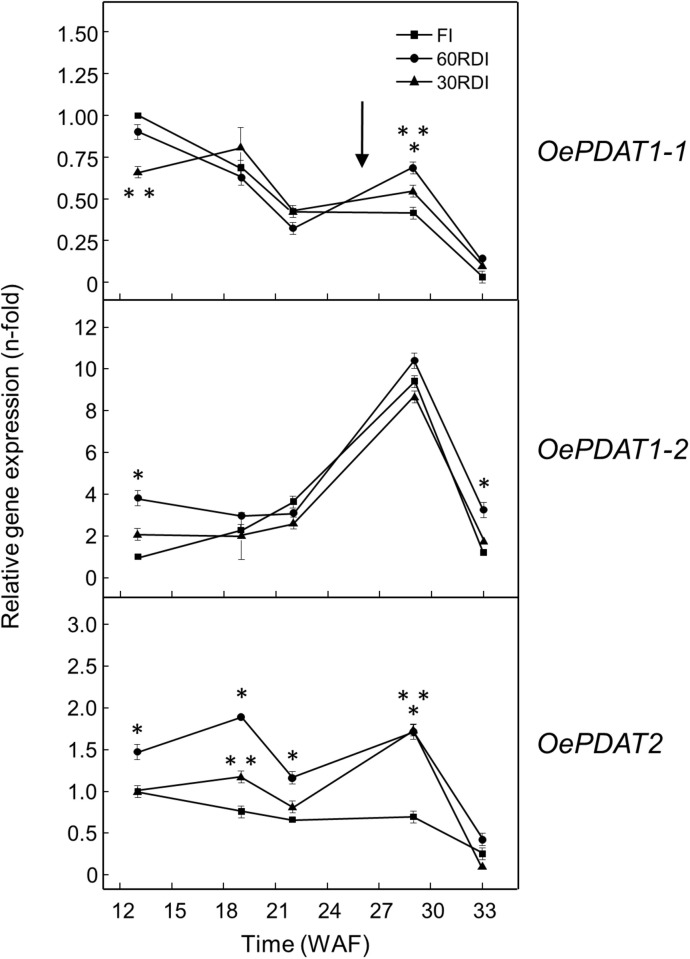
Effect of regulated deficit irrigation treatments on the relative expression levels of olive *PDAT* genes in the mesocarp tissue from cultivar Arbequina during olive fruit development and ripening. The relative expression levels were determined by qRT-PCR at the indicated stages of fruit development as described in “Materials and Methods,” using the expression level of the corresponding gene at 13 WAF from FI treatment as calibrator. Data are presented as means ± SD of three biological replicates. ^∗^Indicates that 60 RDI is significantly different (*P* < 0.05) to FI by two-way ANOVA with a Bonferroni post-test. ^∗∗^Indicates that 30 RDI is significantly different (*P* < 0.05) to FI by two-way ANOVA with a Bonferroni post-test.

Several studies have evaluated the effect of different water regimes on olive oil yield and composition (Fernández, 2014; Gonçalves et al., 2020). One of the most intriguing aspects when applying RDI strategies in olive is that a significant reduction of irrigation is not usually proportionally reflected in the reduction of oil yield (Iniesta et al., 2009; Hernandez-Santana et al., 2017). The tolerance of olive fruit to water stress has been explained by the lower sensitivity of the oil synthesis processes to water deficit than other processes like vegetative growth (Hernandez-Santana et al., 2017). Previous work from our group studied the effect of three different RDI treatments on the Arbequina mesocarp oil content, fatty acid composition, and fatty acid desaturase gene expression levels (Hernández et al., 2018). 60RDI and 30RDI treatments, which produced substantial levels of water stress, did not cause significant differences in mesocarp oil content between RDI and FI treatments, except a small decrease detected at the initial stages of mesocarp development. In line with those observations, in the present work, no significant alterations were found in the transcript levels of any of the olive *PDAT* genes when comparing RDI and FI treatments, apart from a slightly higher expression level for *OePDAT1-1* and *OePDAT2* in the case of the 60RDI and 30RDI treatments at the onset of the ripening period (Figure 6). On the contrary, a strong increase and decrease in the expression levels of *CsPDAT2-A* and *CsPDAT2-B* genes, respectively, have been reported in *Camelina sativa* seedlings in response to drought treatment (Yuan et al., 2017). These contrasting results could suggest the existence of species-dependent transcriptional mechanisms affecting *PDAT* genes in the water stress response.

### Transcriptional Regulation of Phospholipid:Diacylglycerol Acyltransferase Genes in the Olive Fruit Mesocarp in Response to Abiotic Stresses

To analyze the effect of distinct abiotic stresses on the transcript levels of the olive *PDAT* genes in Picual and Arbequina mesocarp tissue, olive tree branches with olive fruit at turning stage (28 WAF) were incubated for 24 h altering the standard conditions (25°C with 12 h light/12 h dark cycle) dependent on the effect to be examined. No significant changes in the oil content were observed in the fruit mesocarp during the stress treatments, very likely because the timescale involved (24 h) may be too short to observe that effect (Supplementary Figures 2, 3). In addition, no substantial variations in the expression levels of olive *PDAT* genes were detected in the mesocarp tissue when olive fruit were incubated under the above-mentioned standard conditions (Supplementary Figure 4).

**FIGURE 7:**
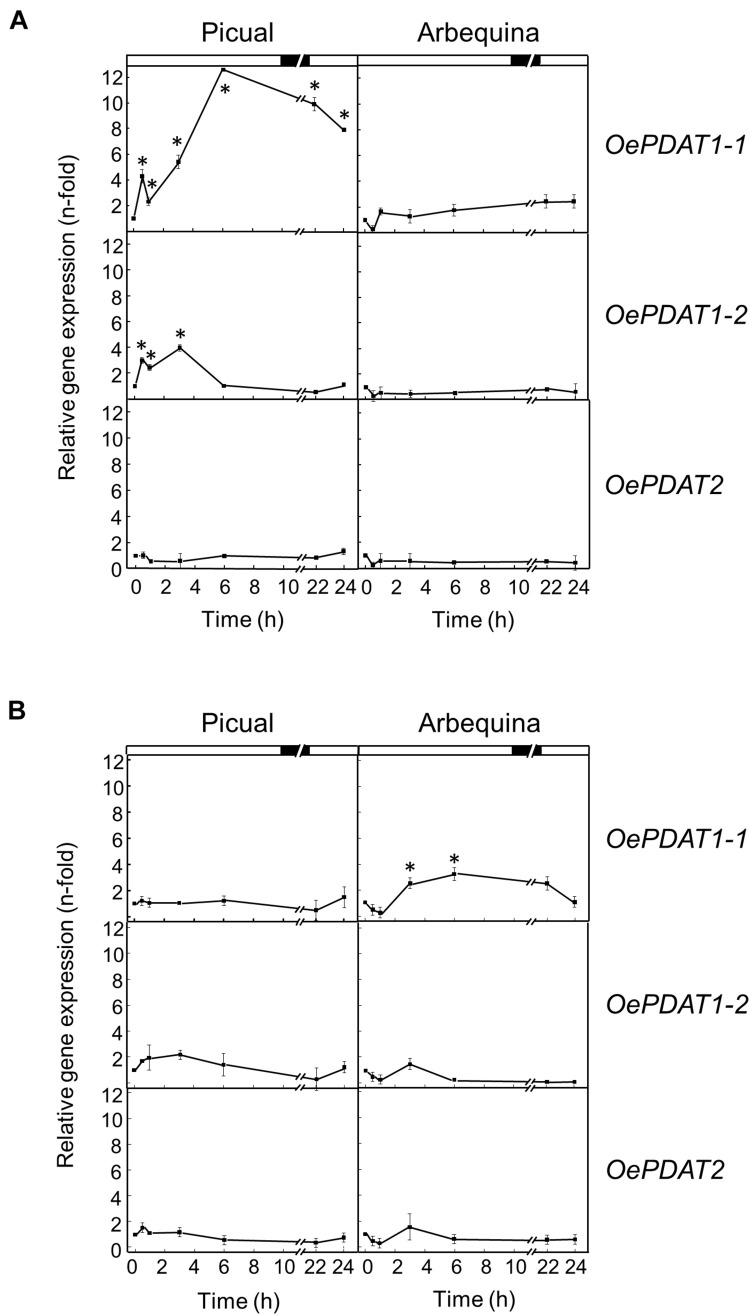
Effect of low temperature **(A)** and high temperature **(B)** on the relative expression levels of olive *PDAT* genes in the mesocarp tissue from Picual and Arbequina cultivars. Olive tree branches with about 100 olive fruit (28 WAF) were incubated using standard conditions except that the temperature was 15°C **(A)** or 35°C **(B)**. At the indicated times, the relative expression levels were determined by qRT-PCR as described in “Materials and Methods,” using the expression level of the corresponding gene at zero time as calibrator. Data are presented as means ± SD of three biological replicates. ^∗^Indicates significantly different (*P* < 0.05) to time 0 h by two-way ANOVA with a Bonferroni post-test. Boxes in the upper part indicate light (open) or dark (closed) periods.

The incubation at low temperature (15°C) of olive fruit only caused a strong increment of 12-fold in *OePDAT1-1* of cultivar Picual transcript levels during the first 6 h of treatment, keeping high values the rest of the time of the experiment (Figure 7A). These results indicate a cultivar-dependent differential transcriptional response to cold of the olive *PDAT1-1* gene, as it has been reported for the olive *FAD2* genes (Hernández et al., 2020b). A similar up-regulation has been described for *CsPDAT1-A* and *CsPDAT1-C* genes in *Camelina sativa* seedlings under cold stress (Yuan et al., 2017). Regarding high temperature, when olive fruit was incubated at 35°C the transcript levels of the three olive *PDAT* genes remained almost unaffected (Figure 7B). Interestingly, it has been recently reported that heat stress stimulates *AtPDAT1* expression in rosettes of Arabidopsis seedlings (Demski et al., 2020). Furthermore, AtPDAT1-mediated TAG accumulation in Arabidopsis seedlings has been found to increase heat resistance and augments basal thermotolerance (Mueller et al., 2017). All these data indicate that PDAT plays a role in the response and adaptation of plants to temperature alterations.

**FIGURE 8:**
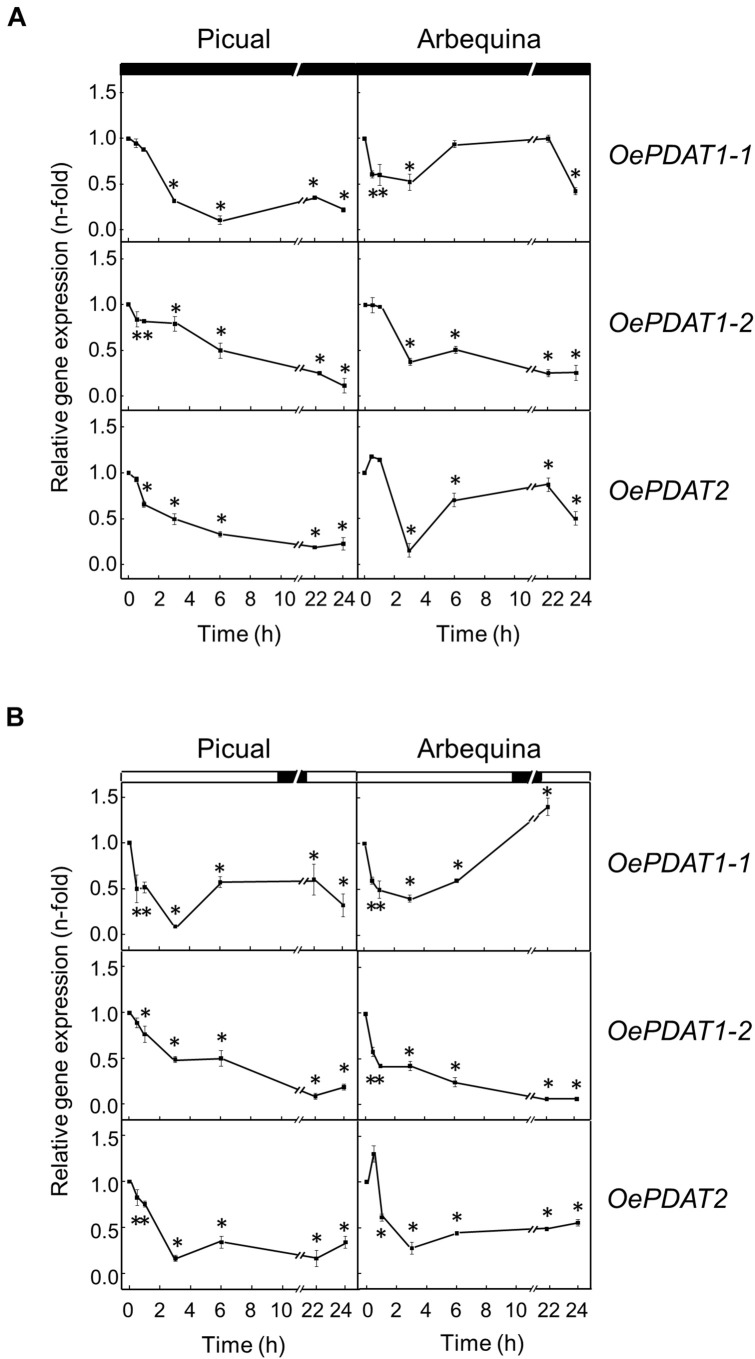
Effect of darkness **(A)** and wounding **(B)** on the relative expression levels of olive *PDAT* genes in the mesocarp tissue from Picual and Arbequina cultivars. Olive tree branches with about 100 olive fruit (28 WAF) were incubated using standard conditions except that the olive fruit were incubated under darkness **(A)** or were mechanically damaged **(B)**. At the indicated times, the relative expression levels were determined by qRT-PCR as described in “Materials and Methods,” using the expression level of the corresponding gene at zero time as calibrator. Data are presented as means ± SD of three biological replicates. ^∗^Indicates significantly different (*P* < 0.05) to time 0 h by two-way ANOVA with a Bonferroni post-test. Boxes in the upper part indicate light (open) or dark (closed) periods.

To examine whether darkness affects *PDAT* expression levels in the olive mesocarp from Picual and Arbequina cultivars, olive branches were incubated at 25°C for 24 h in the darkness. Expression analysis showed a fast and strong decline of *OePDAT1-1*, *OePDAT1-2*, and *OePDAT2* transcript levels in both cultivars mostly during the first 3 h of treatment, remaining with small values the rest of the experiment (Figure 8A). Interestingly, the inactivation by darkness of the acetyl-CoA carboxylase enzyme, which catalyzed the first committed step of *de novo* fatty acid synthesis in plants, has been reported in Arabidopsis (Ye et al., 2020), which is in accordance with the observed down-regulation of olive *PDAT* genes. Additionally, in agreement with our results, Fan et al. (2017) reported that increased TAG accumulation in Arabidopsis leaves by overexpression of *AtPDAT1* before prolonged dark treatment enhanced oxidative stress and dark-induced cell death. These data indicate that PDAT is not involved in plant survival under extended darkness.

Finally, the effect of wounding on the expression levels of olive *PDAT* genes was investigated in the olive mesocarp from Picual and Arbequina cultivars subjected to mechanical damage from branches incubated at standard conditions. First, it was confirmed that the wounding treatment of the mesocarp tissue was properly carried out because olive 13-lipoxygenase and 13-hydroperoxide lyase genes, which have been previously shown to be wound-inducible in plant tissues (Padilla et al., 2014), were transiently induced (Supplementary Figure 5 and Supplementary Table 1). In both cultivars, *OePDAT1-1*, *OePDAT1-2*, and *OePDAT2* transcript levels rapidly and markedly decreased during the first 3 h after wounding, keeping at low levels the rest of the incubation (Figure 8B). The observed down-regulation of olive *PDAT* gene expression in response to wounding could be explained to avoid the channeling of linoleic and linolenic acids from PC to be accumulated in TAG since both polyunsaturated fatty acids can be transformed to lipid peroxides that may act as antimicrobial compounds (Wang et al., 1998), or they could act as precursors via the lipoxygenase pathway of oxylipins, which constitute signal molecules involved in plant defense (Wasternack and Feussner, 2018). Accordingly, the transient induction of several *FAD2* genes has been described in the olive mesocarp in response to wounding (Hernández et al., 2011, 2020b).

## Conclusion

The isolation and characterization of three olive *PDAT* genes have been carried out. Sequence analysis of these genes (*OepPDAT1-1*, *OepPDAT1-2*, and *OepPDAT2*) indicates that they code for PDAT enzymes. Transcript profiling shows a spatial and temporal regulation of the expression levels of the *PDAT* genes in the olive fruit during development and ripening and, together with the pattern of oil accumulation suggest that, in addition to *DGAT* genes, *OePDAT1-1* could participate in the TAG synthesis in the seed, while *OePDAT1-2* may contribute to the TAG content in the mesocarp and, therefore, in the olive oil. These data also indicate that the relative contribution of PDAT and DGAT enzymes to TAG synthesis in olive seems to be organ and tissue-dependent. Moreover, the expression of *PDAT* genes in the olive fruit is regulated by water regime, temperature, light, and wounding, indicating that PDAT is involved in the response to a range of relevant abiotic stresses. This research constitutes a significant step to elucidate the factors controlling the oil content and accumulation in oil fruit. With regard to olive, this information will help to design molecular markers for the marker-assisted selection of novel olive cultivars with increased oil content.

## Data Availability Statement

The original contributions presented in the study are included in the article/Supplementary Material, further inquiries can be directed to the corresponding author/s.

## Author Contributions

MLH managed and performed the harvest of plant material and the stress experiments, and carried out RNA isolation, cDNA synthesis, and oil content determination. SM, MDS, ÚG, and AP performed the gene cloning and qRT-PCR analysis. LS revised the study and the manuscript. JM-R conceived and designed the study and wrote the manuscript. All authors discussed, commented, and approved the final version of the manuscript.

## Conflict of Interest

The authors declare that the research was conducted in the absence of any commercial or financial relationships that could be construed as a potential conflict of interest.

## Publisher’s Note

All claims expressed in this article are solely those of the authors and do not necessarily represent those of their affiliated organizations, or those of the publisher, the editors and the reviewers. Any product that may be evaluated in this article, or claim that may be made by its manufacturer, is not guaranteed or endorsed by the publisher.

## Footnotes

1
http://www.ncbi.nlm.nih.gov/structure/cdd/wrpsb.cgi

2
https://pfam.xfam.org/

3
http://www.cbs.dtu.dk/services/NetNGlyc/

4
http://www.cbs.dtu.dk/services/TMHMM/

5
http://www.softberry.com

6
http://www.cbs.dtu.dk/services/TargetP/

7
http://bioinfo.ut.ee/primer3/
