# Supplementary material for: Distinct Physiological Roles of Three Phospholipid:Diacylglycerol Acyltransferase Genes in Olive Fruit with Respect to Oil Accumulation and the Response to Abiotic Stress
Source: Front Plant Sci. 2021 Nov 12;12:751959. doi: 10.3389/fpls.2021.751959 (PMC8632719; doi:10.3389/fpls.2021.751959)
Supplement: Supplementary file 1 [file Presentation_1.PDF]

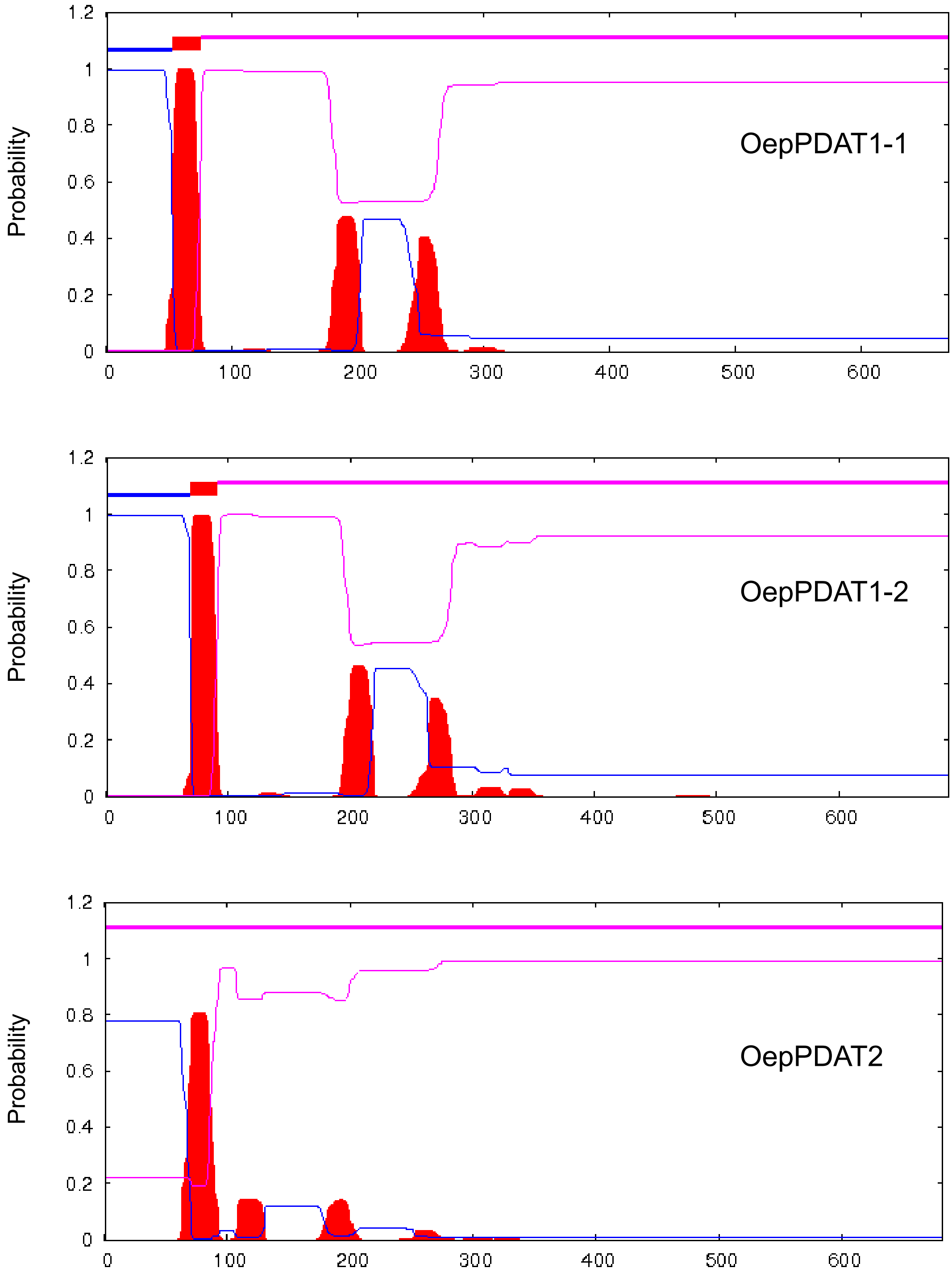

Fig. S1. Predicted transmembrane domain for olive PDAT sequences. A single predicted transmembrane helix (shown in red) was identified by TMHMM analysis for OepPDAT1-1 and OepPDAT1-2. Regions of the olive PDAT sequences predicted to be located inside or outside the membrane are shown in blue and pink, respectively.

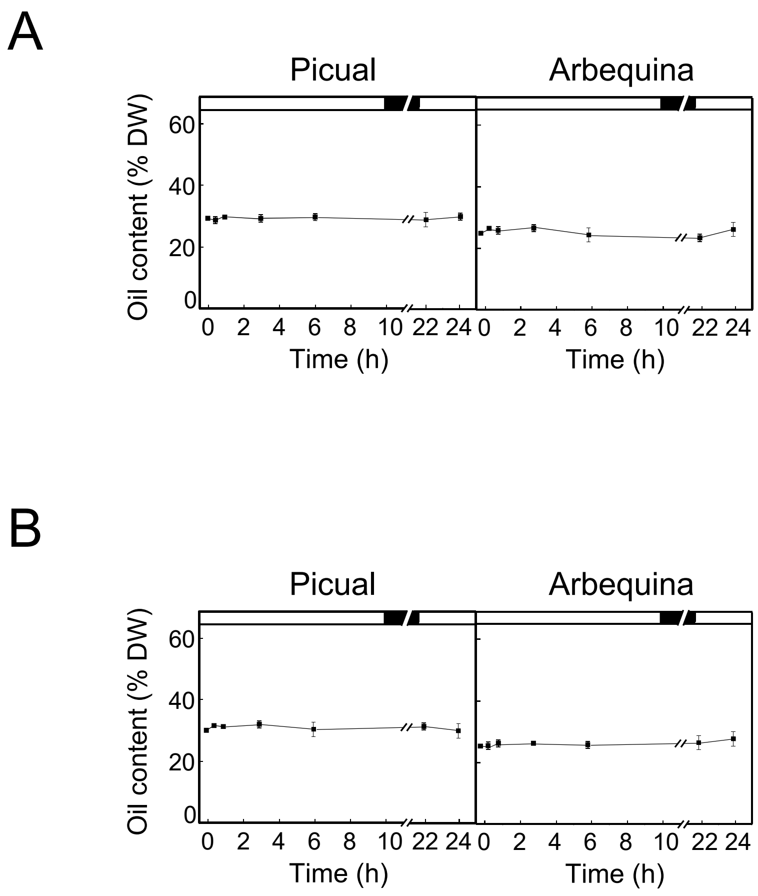

Fig. S2. Effect of low temperature (A) and high temperature (B) on the oil content of mesocarp tissue from Picual and Arbequina cultivars. Olive tree branches with about 100 olive fruit (28 WAF) were incubated using standard conditions except that the temperature was 15 ºC (A) or 35 ºC (B). At the indicated times, the oil content was determined as described in Materials and methods. Data are presented as means ±SD of three biological replicates. *Indicates significantly different ($P < 0.05$) to time 0 h by two-way ANOVA with a Bonferroni post-test. Boxes in the upper part indicate light (open) or dark (closed) periods.

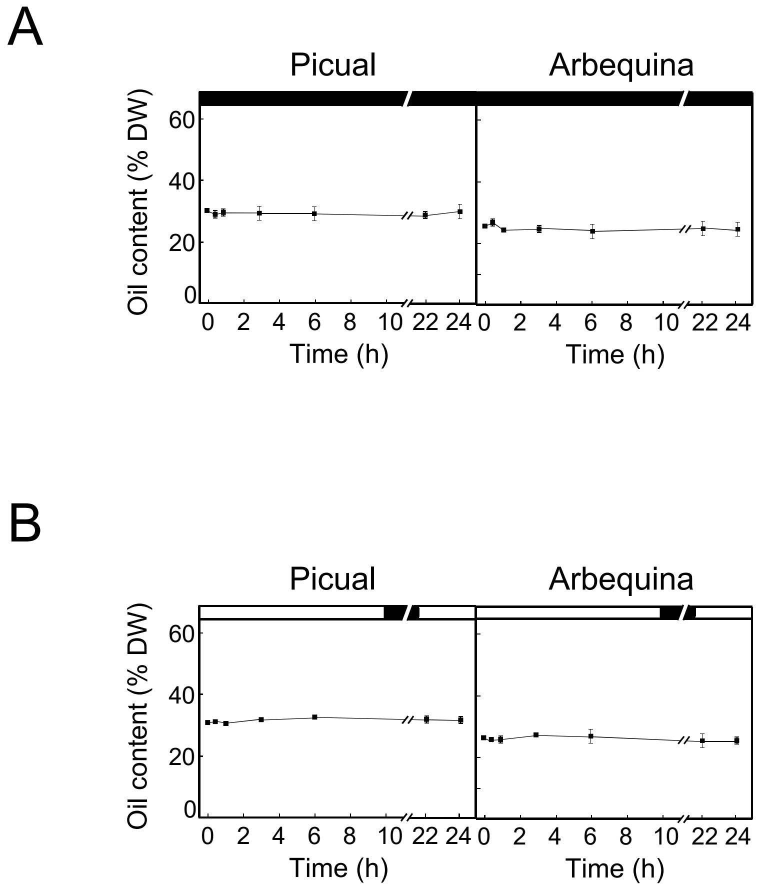

Fig. S3. Effect of darkness (A) and wounding (B) on the oil content of the mesocarp tissue from Picual and Arbequina cultivars. Olive tree branches with about 100 olive fruit (28 WAF) were incubated using standard conditions except that the olive fruit were incubated under darkness (A) or were mechanically damaged (B). At the indicated times, the oil content was determined as described in Materials and methods. Data are presented as means ±SD of three biological replicates. *Indicates significantly different ($P < 0.05$) to time 0 h by two-way ANOVA with a Bonferroni post-test. Boxes in the upper part indicate light (open) or dark (closed) periods.

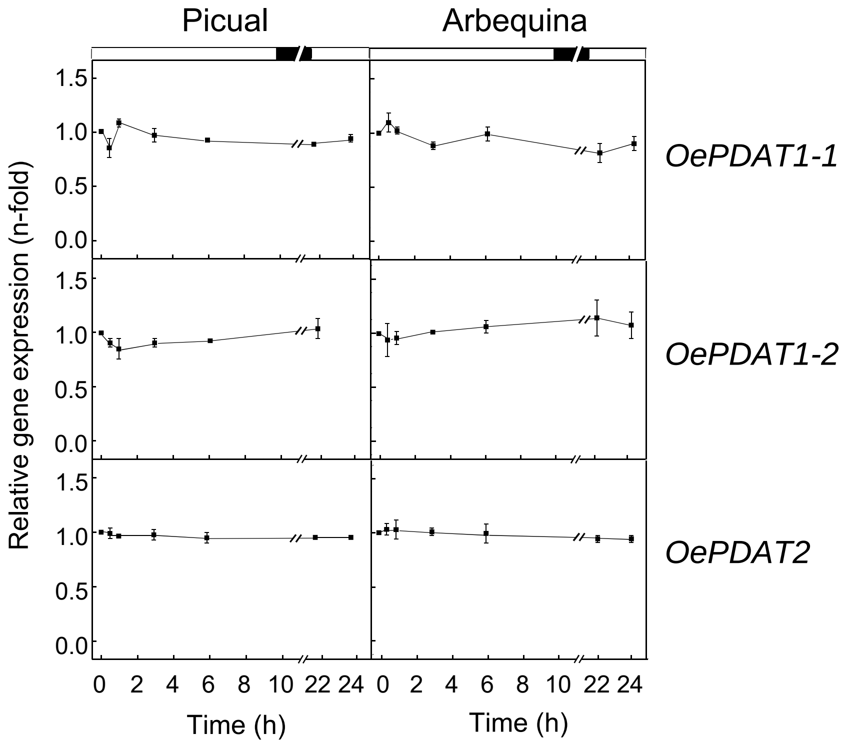

Fig. S4. Effect of incubation under the standard conditions on the relative expression levels of olive *PDAT1-1, PDAT1-2,* and *PDAT2* genes in the mesocarp tissue from Picual and Arbequina cultivars. Branches with about 100 olive fruit (28 weeks after flowering) were incubated using standard conditions. At the indicated times, relative expressions levels were determined by qRT-PCR as described in Materials and methods, using the expression level of the corresponding gene at zero time as calibrator. Data are presented as means ± SD of three biological replicates.

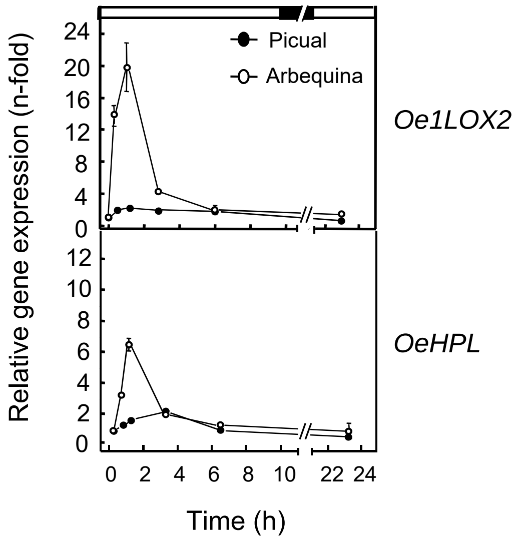

Fig. S5. Effect of wounding on the relative expression levels of olive *1LOX2* and *HPL* genes in the mesocarp tissue from Picual and Arbequina cultivars. Branches with about 100 olive fruits (28 weeks after flowering) were incubated using standard conditions except that olive fruit were mechanically damaged at zero time. At the indicated times, relative expression levels were determined by qRT-PCR as described in Materials and methods, using the expression level of the corresponding gene at zero time as calibrator. Data are presented as means ± SD of three biological replicates.
