# Supplementary material for: Distinct Physiological Roles of Three Phospholipid:Diacylglycerol Acyltransferase Genes in Olive Fruit with Respect to Oil Accumulation and the Response to Abiotic Stress
Source: Front Plant Sci. 2021 Nov 12;12:751959. doi: 10.3389/fpls.2021.751959 (PMC8632719; doi:10.3389/fpls.2021.751959)
Supplement: Supplementary file 2 [file Table_1.PDF]

Table S1. Sequences of the primers pairs used for gene expression analysis by qRT-PCR in the present study.

Gene	Sequence	Amplicon size (bp)	Reference
OepPDAT1-1	Forward: 5’-AATTGCTGCTGGGGCTAAG-3’<br>Reverse: 5’-CCCGGCTCAAGTTATAGAGGT-3’	105	Hernández et al. (2020a)
OepPDAT1-2	Forward: 5’-TCAAATGGTCGGAAAGGATT-3’<br>Reverse: 5’-TTTGGGGAATATGGTTGACAC-3’	143	Hernández et al. (2020a)
OepPDAT2	Forward: 5’-CGGGAAGTAGCAAAGGAGAG-3’<br>Reverse: 5’-GCATTCAAACATCCCCATTAG-3’	106	This work
Oep1LOX2	Forward: 5’-GAGAATTGGGTGCGTTCATAC-3’<br>Reverse: 5’-TCCTCTGGTGTGGCTAATGTC-3’	158	Padilla et al. (2014)
OepHPL	Forward: 5’-TCTCATACCCTTTCTGGCTTG-3’<br>Reverse: 5’-ATCGTAAACCCACCAAATGC-3’	175	Padilla et al. (2014)
OeUBQ2	Forward: 5’-AATGAAGTCTGTCTCTCCTTTGG-3’<br>Reverse: 5’-AAGGGAAATCCCATCAACG-3’	132	Hernández et al. (2009)
